# Development and validation of a predictive model for frailty risk in older adults with cardiovascular-metabolic comorbidities

**DOI:** 10.3389/fpubh.2025.1561845

**Published:** 2025-04-22

**Authors:** Lulu Yan, Entong Ren, Chenjiao Guo, Yuanyuan Peng, Hao Chen, Weihua Li

**Affiliations:** ^1^Health Science Center, Yangtze University, Jingzhou City, China; ^2^Department of Nursing, General Hospital of Southern Theatre Command of PLA, Guangzhou, Guangdong, China; ^3^Department of Medicine, Guangdong Pharmaceutical University, Guangzhou, Guangdong, China

**Keywords:** predictive model, frailty, cardiovascular-metabolic comorbidities, CHARLS, cross-sectional study

## Abstract

**Background:**

With the rapid progression of population aging, the number of frail individuals is steadily rising, making frailty a pressing public health issue that demands urgent attention. Compared to individuals with a single cardiovascular-metabolic disease, patients with cardiovascular-metabolic multimorbidity (CMM) are more prone to developing frailty. This study aimed to develop and validate a predictive model for assessing frailty risk in older adult patients with CMM.

**Methods:**

The data came from participants in the 2015 wave of the China Health and Retirement Longitudinal Study (CHARLS). The study population comprised individuals aged 60 years and older with CMM and complete frailty scale measurements. Frailty status was evaluated using the Fried Frailty Scale. 26 indicators, including socio-demographic characteristics, lifestyle factors, overall health condition, and psychological well-being. The entire sample was randomly allocated into training and validation sets at a 7:3 ratio. LASSO regression and logistic regression was conducted to evaluate factors associated with frailty. A nomogram was constructed using the identified predictors to predict outcomes. The discrimination, accuracy, and clinical effectiveness of the model were evaluated by the area under the receiver operating characteristic curve (AUC), calibration plot, and decision curve analysis (DCA).

**Results:**

The study included 2,164 older adult CMM participants, with 387 (17.88%) displaying frailty symptoms. Binary logistic regression analyses revealed that depression, social activity, history of falls, life satisfaction, ADL scores, cognitive function, age and the number of CMDs were significantly associated with frailty. These eight factors were incorporated into the nomogram model, and the AUC values for the predictive model were 0.816 (95% CI = 0.787–0.848) and 0.816 (95% CI = 0.786–0.846) for the training and validation sets, respectively, indicating effective discrimination. Hosmer-Lemeshow test results showed *p* = 0.073 and *p* = 0.245 (both > 0.05), with calibration curves indicating strong alignment between the model’s predictions and actual outcomes. The DCA demonstrated the model’s substantial clinical utility.

**Conclusion:**

The nomogram prediction model developed in this research is a reliable and effective tool for assisting clinicians in identifying frailty in older adult CMM patients at an early stage, providing a scientific foundation for individualized health management and intervention.

## 1 Introduction

With the acceleration of population aging and the extension of life expectancy, disability, mortality, and disease burden contribute to making cardiovascular-metabolic diseases (CMD) and cardiovascular-metabolic comorbidities (CMM) major public health challenges worldwide (1, 2). CMM is one of the most common and stable comorbidity clustering patterns (3), referring to individuals affected by two or more CMD simultaneously. In this study, CMD primarily include hypertension, dyslipidemia, diabetes, heart disease, and stroke (4, 5). 2023 World Heart Report, the global prevalence and mortality of cardiovascular diseases have continuously increased over the past three decades as population aging intensifies. Compared to patients with a single chronic disease, those with CMM experience higher levels of mentalstress (6) and cognitive decline (7), along with a reduction in life expectancy by 15 years (8). Additionally, they face a greater healthcare burden (9) and an increased mortality risk (2). CMM imposes substantial burdens on patients, families, and the healthcare system, emerging as a major public health issue that adversely affects the health and quality of life of individuals in China. Moreover, studies have shown that the prevalence of frailty is higher among older adults with CMM than among those with a single type of CMD (10). Thus, strengthening health management for CMM is essential.

Frailty is a complex multidimensional condition, and related studies based on the China Health and Retirement Longitudinal Study (CHARLS) database from 2011 to 2015 found that the incidence of frailty among older adults ranged from 8.9% to 28.4% (11, 12). Similarly, research conducted across 10 countries in Southern and Northern Europe has reported a frailty prevalence of 17% among older adults (13), indicating a high global incidence of frailty in this population. The main characteristics of frailty are a significant reduction in physiological reserves and a decrease in the organism’s ability to repair itself, a state that causes individuals to have a lower adaptive capacity when faced with external physiological or psychological stress (14). Existing studies have identified frailty as a predictor of mortality, falls, disease progression, and hospital readmission among older adults (15). The 2020 International Consensus on Frailty guidelines emphasize that frailty significantly increases the risk of adverse health outcomes, including emergency hospitalizations, cognitive impairment, depression, suicidal behaviors, and even death. Moreover, frail individuals incur healthcare costs that are 22% higher than those of their non-frail counterparts (16). Consequently, frailty significantly endangers the physical and mental health. As well as the economic well-being, of older adults (17, 18). Comparative studies on frailty among older adults across different countries have demonstrated that variations in frailty prevalence are influenced by differences in education, cultural background, and economic conditions (13, 19, 20). These findings highlight the necessity of developing tailored intervention strategies based on the specific contexts of each country.

Risk prediction models serve as essential tools for assessing frailty risk, enabling the early identification of high-risk populations, optimizing the allocation of public health resources, and facilitating the development of personalized intervention strategies. Existing research primarily focuses on frailty and its determinants in patients with a single disease, such as cardiovascular or cerebrovascular diseases, while studies specifically addressing frailty in CMM patients remain limited. The complexity and multidimensional nature of CMM comorbidity patterns make it difficult for single-disease studies to comprehensively elucidate the mechanisms and influencing factors of frailty. Studies have indicated that CMM and frailty are two interrelated conditions in older adults (20). Numerous shared biological, metabolic, and physiological mechanisms exist between CMM patterns and frailty, which intertwine and contribute to the onset and progression of frailty (21). Their close relationship often leads to coexistence, with each condition reinforcing the other, creating a vicious cycle that results in worsened patient outcomes (22). Since the early development of frailty is a dynamic and potentially reversible process (21), the National Institute for Health and Care Excellence (NICE) and the British Geriatrics Society have emphasized the importance of frailty identification to recognize CMM patients at high risk of adverse outcomes who may benefit from timely interventions (17). Therefore, this study utilizes data from the CHARLS to develop and validate a frailty prediction model for older adults with CMM by integrating sociodemographic characteristics, lifestyle factors, health status, and psychological conditions. This model not only facilitates a comprehensive understanding of the specific characteristics of frailty in the Chinese older adult CMM population but also enhances precision in health management, reduces healthcare burdens, and enables targeted prevention strategies for high-risk individuals to slow frailty progression. Consequently, it holds significant clinical and public health implications.

## 2 Materials and methods

### 2.1 Study design and population

This study utilizes follow-up data from the 2015 wave of the CHARLS, organized and conducted by the Research Center for Healthy Aging and Development and the National School of Development at Peking University. The study covered 23 provinces and municipalities (including county-level cities) in China, representing 85% of the country’s population, which provides a robust representation of the population (23). CHARLS study protocol received ethical clearance from the Biomedical Ethics Committee at Peking University (IRB00001052-11015), all participants signed the informed consent form upon access.

The criteria for inclusion in this study were set as follows: (1) age≥60 years; (2) diagnosis of CMM. Conversely, the exclusion criteria included: (1) more than 20% of the data for individual variables were missing; (2) incomplete or invalid frailty scale data. Based on these criteria, Overall 2,164 participants were included in the study.

### 2.2 Data collection

#### 2.2.1 Definition of frailty

The concept of frailty was initially put forward by Fried (24). At present, the Fried Frailty Scale is the most commonly utilized instrument for evaluating frailty. Due to its strong predictive validity, it is frequently utilized in both research and clinical settings to measure frailty (22). The thresholds for reduced grip strength and slowed walking speed were determined based on previous frailty studies and literature utilizing CHARLS data (25). In this research, frailty is defined as a binary outcome variable, with scores ranging from 0 to 5. Each item was scored as “1” or “0” (yes or no), with a total score of ≥3 indicating frailty. The assessment of the five key items is detailed below.

##### 2.2.1.1 Weight loss

A reduction in weight of 5 kg or more compared to the prior year, or a current BMI of ≤ 18.5 kg/m^2^ (15).

##### 2.2.1.2 Loss of grip strength

Measured by standing and grasping a grip strength meter at a right angle to the elbow, holding it firmly for a few seconds to maximize grip strength. Missing values were defined if one or both hands had undergone surgery, or if swelling, inflammation, severe pain, or injury had occurred in the last 6 months. Grip strength was considered below normal if it fell below 20% of the sex and BMI quartile-adjusted weighted population distribution (25).

##### 2.2.1.3 Reduced walking pace

Determined by the duration to traverse 2.5 meters, with the average of two measurements taken. If there was recent surgery or trauma, this was considered a missing value. Walking speed was considered below normal if it fell below 20% of the weighted population distribution, adjusted for sex and height (25).

##### 2.2.1.4 Low physical activity

Physical activity was evaluated using the relevant item from the questionnaire. “Do you consistently walk for at least 10 min per week?” This variable differs from the Fried scale but has been used in similar studies to assess physical activity in frailty, demonstrating its applicability and reliability in evaluating frailty (26).

##### 2.2.1.5 Fatigue

Measured by the Center for Epidemiologic Studies-Depression scale (CES-D) from the Questionnaire of Health Status and Functioning (QHSF). The questions “I find it hard to do anything” and “I feel like I cannot go on with my life” were answered by the patient as “Most or all of the time” or “Occasionally or moderately,” indicating the participant is fatigued (24).

#### 2.2.2 Independent variables

This study identified 26 potential predictors of frailty, classified into four key domains: (1) sociodemographic factors; (2) lifestyle; (3) health status; and (4) psychological conditions. The selection of variables was guided by three principles: (1) Documentary evidence: variables were selected based on their significance and interpretability in previous frailty research. For example, factors such as age, gender, and sleep duration have been consistently identified as critical predictors in multiple frailty prediction models (26–28). (2) Biological mechanisms: variables directly linked to frailty pathophysiology were incorporated, including depression (associated with chronic inflammation and neuroendocrine dysregulation), smoking (which impairs muscle metabolism), and the ADL score (indicative of functional decline). (3) Multidisciplinary expert consultation: Experts in public health, pharmacy, statistics, and geriatric medicine were consulted to validate the rationale for variable selection. In this study, the specific characteristics of each theme are outlined below, and the assignment of categorical variables is detailed in Table 1.

**Table 1:** Categorical variable assignment.

Variable	Variable assignment
Sex	Male = 1; female = 0
Physical disability	Yes = 1; no = 0
Depression	Yes = 1; no = 0
Smoke	Yes = 1; no = 0
Drink	Yes = 1; no = 0
Social activity	Yes = 1; no = 0
Be vexed by pain	Yes = 1; no = 0
Fall down	Yes = 1; no = 0
Sleep quality	Yes = 1; no = 0
Life satisfaction	Yes = 1; no = 0
Residence	Urban = 1; rural = 0
Number of CMDs	2 ~ 3 = 0; 4 ~ 5 = 1
Age	60 ~ 69 = 1; 70 ~ 79 = 2; ≥80 = 3
Child satisfaction	Yes = 1; no = 2; No child = 3
Distant object vision	Good = 1; common = 2; bad = 3
Near object vision	Good = 1; common = 2; bad = 3
Hearing	Good = 1; common = 2; bad = 3
Memory	Good = 1; common = 2; bad = 3
Education	Below junior high school = 1; high school or vocational school = 2; college or above = 3
Financial resources after retirement	Children = 1; savings or pension = 2; else = 3
Marriage	Married = 1; divorced or widowed = 2; unmarried = 3

The following socio-demographic factors were considered: age (60–69, 70–79 and ≥80); sex (female, male); education level (below junior high, high school or vocational school, and university level or higher); marital status (married, divorced or widowed, and unmarried); Body Mass Index (BMI) is calculated by dividing an individual’s weight in kilograms by the square of their height in meters; place of residence (rural, urban); income level (total household income minus total expenditures); and post-retirement financial resources (children, personal savings or business insurance, and other).

Lifestyle factors include sleep quality (categorized as either good or poor based on participants’ responses); sleep duration (determined by the average number of hours participants report sleeping over the past month); socialization (yes, no); smoking (yes, no); drinking (yes, no).

Overall health condition includes ADL (Activities of Daily Living) scores; near vision (good, fair, and poor); distance vision (good, fair, and poor); hearing (good, fair, and poor); history of falls (yes, no); disability (yes, no); memory (good, fair, and poor); pain (yes, no); and the number of CMDs (2–3, 4–5). The Katz ADL Independence Index (29) was utilized to evaluate daily living activities, which encompasses six items: eating, dressing, bathing, toileting, urinary or fecal control, and transferring in or out of bed. Results were categorized as follows: A score of 1 is assigned for no difficulty or completing despite difficulty, and a score of 0 is assigned for requiring assistance due to difficulty or inability to complete. Higher scores on the scale indicate better patient self-care abilities.

Psychological status includes depression score (yes, no), life satisfaction (satisfied, dissatisfied), child satisfaction (satisfied, dissatisfied, and childless), and cognitive function. Depression was assessed using the CESD-10 scale, which consists of 8 positive items and 2 negative items. Each item is scored as follows: 0 points for “rarely or never”; 1 point for “infrequently”; 2 points for “sometimes” or “approximately half of the time”; and 3 points for “most of the time.” The total score varies between 0 and 30. A score above 10 indicates the presence of depression (30), and the depression outcome was categorized as yes or no. Cognitive function in CHARLS was assessed in three areas: the Telephone Interview for Cognitive Status (TICS-10), word recall, and drawing. The TICS-10 included questions about the season, day of the week, year, month, and date, scoring 0 for incorrect responses and 1 for correct ones. Participants were also asked to subtract 7 from 100, repeating the subtraction five times. Each correct answer earned 1 point, primarily assessing attention and calculation ability. For word recall, participants were presented with a list of 10 words and then asked to recall them soon after. They were then given additional questions before recalling the words again, with 1 point for each correct word, and the average of the two recalls was taken. For drawing, Participants were shown an image of two overlapping five-pointed stars and instructed to reproduce it onto a blank sheet of paper. A correct drawing earned 1 point, and an incorrect drawing received 0 points. This assessed visuospatial ability. The total score varied between 0 and 21, with lower scores reflecting worse cognitive function (31).

### 2.3 Data analysis

This research utilized information sourced from the 2015 CHARLS dataset. Categorical data were presented in terms of percentages. To compare groups, either the χ^2^ test or Fisher’s exact test was applied. For continuous variables, medians along with interquartile ranges were used to summarize the data, and the Mann–Whitney U test was employed to assess differences between groups. The maximum proportion of missing values for any independent variable was limited to 20%. In this study, the Multiple Imputation method was employed to estimate missing values by creating multiple complete datasets. We assume that the missing data is Missing at Random (MAR), meaning that the occurrence of missing values may be related to some observed variables. To assess the validity of the imputation, a sensitivity analysis was performed, comparing the results of the imputed dataset with the original dataset, to ensure that the imputation method did not significantly affect the study’s conclusions. To ensure the stochasticity and replicability of the sampling methodology, random seeds were set in Rstudio (32), and the total sample was systematically divided into a training set (*n* = 1,514) and a validation set (*n* = 650) in a 7:3 ratio.

A nomogram was utilized to portray the risk factors associated with frailty among older adult patients suffering from CMM. Least Absolute Shrinkage and Selection Operator (LASSO) regression analysis was carried out on the training set in order to pinpoint the significant risk factors related to frailty. To determine the optimal tuning parameter (*λ*) for the LASSO regression, 10-fold cross-validation was conducted. The LASSO algorithm was applied to identify the most significant predictors, effectively handling high-dimensional data and multicollinearity, while enhancing model interpretability through variable selection. The identified significant predictors were then analyzed using binary logistic regression, and variables with a *p*-value<0.05 were incorporated into the nomogram model.

The model’s ability to distinguish between outcomes was measured by calculating the area under the receiver operating characteristic (ROC) curve, commonly referred to as AUC. To gauge how well the predicted probabilities aligned with actual results, calibration curves were utilized. Additionally, decision curve analysis (DCA) was performed to evaluate the model’s practical utility in a clinical setting. All statistical analyses were carried out using R software (version 4.4.2) and SPSS (version 25.0). Two-tailed statistical tests were employed, with a *p*-value < 0.05 considered statistically significant.

## 3 Result

### 3.1 Participant characteristics

**Figure 1:**
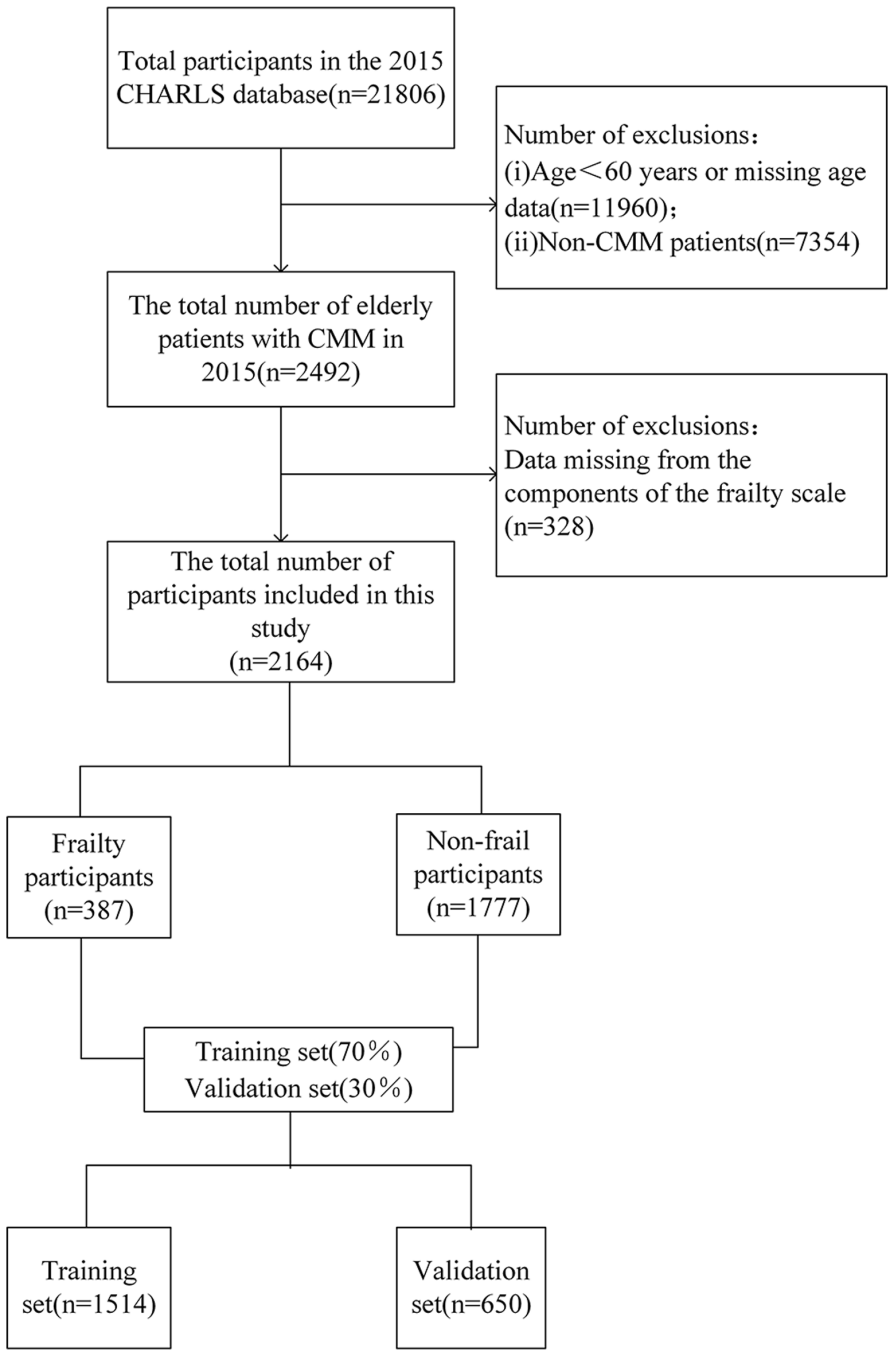
Flowchart of participant selection.

In this study, we ultimately included 2,164 patients. The details of the screening process can be found in Figure 1. The participants in this study ranged in age from 60 to 93 years, with a mean age ± standard deviation of 68.6 ± 6.6 years. The mean age of the frailty group (70.7 ± 7.2 years) was significantly higher than that of the non-frailty group (68.2 ± 6.4 years). Age stratification analysis revealed that the largest proportion of participants were aged 60–69 years (61.5%), followed by those aged 70–79 years (31.0%), while the oldest group (80 years and above) constituted the smallest proportion (7.6%). This distribution pattern suggests that the study population primarily comprises younger older adults (see Supplementary Figure 1). Regarding disease distribution, the study population included 759 individuals with diabetes, 1,294 with dyslipidemia, 1,309 with heart disease, 1847 with hypertension, and 310 with a history of stroke (see Supplementary Figure 2). Among these conditions, the most prevalent comorbidity combination was hypertension and heart disease, and this group also had the highest number of frail individuals (see Supplementary Figure 3).

In this study, 940 were male, accounting for 43.4%, while 1,224 were female, making up 56.6%. A total of 387 patients (17.88%) were classified as frail. The correlation matrix of the 26 predictive factors in this study (see Supplementary Figure 4) demonstrates low correlations among the variables, suggesting a high degree of independence in their ability to predict frailty. Several factors, including age, physical activity, depression, and social activity, showed significant differences (*p*-value < 0.05) between the frail and non-frail groups. The demographic and clinical characteristics of the participants are presented in Table 2. The older adult participants with CMM were systematically assigned to two groups: a training set (*n* = 1,514) and a validation set (*n* = 650), with a 7:3 ratio.

**Table 2:** Frailty prevalence and associated covariates in older adult patients with CMM (*n* = 2,164).

Variable	Total(2,164)	No-frailty(1,777)	Frailty(387)	χ^2^/Z	*p*
Sex(%)				10.117	0.002
Male	940(43.4)	800(45.0)	140(36.2)		
Female	1,224(56.6)	977(55.0)	247(63.8)		
Physical disability(%)				16.184	*p* < 0.01
Yes	1,080(49.9)	851(47.9)	229(59.2)		
No	1,084(50.1)	926(52.1)	158(40.8)		
Depression(%)				115.219	*p* < 0.01
Yes	935(43.2)	673(37.9)	262(67.7)		
No	1,229(56.8)	1,104(62.1)	125(32.3)		
Smoke(%)				0.509	0.520
Yes	431(19.9)	359(20.2)	72(18.6)		
No	1,733(80.1)	1,418(79.8)	315(81.4)		
Drink(%)				14.823	*p* < 0.01
Yes	578(26.7)	505(28.4)	73(18.9)		
No	1,586(73.3)	1,272(71.6)	314(81.1)		
Social activity(%)				56.609	*p* < 0.01
Yes	1,146(53.0)	1,008(56.7)	138(35.7)		
No	1,018(47.0)	769(43.3)	249(64.2)		
Be vexed by pain(%)				22.181	*p* < 0.01
Yes	909(42.0)	705(39.7)	204(52.7)		
No	1,255(58.0)	1,072(60.3)	183(47.3)		
Fall down(%)				121.142	*p* < 0.01
Yes	640(29.6)	436(24.5)	204(52.7)		
No	1,524(70.4)	1,341(75.5)	183(47.3)		
Sleep quality(%)				19.716	*p* < 0.01
Yes	1,280(59.1)	1,090(61.3)	190(49.1)		
No	884(40.9)	687(38.7)	197(50.9)		
Life satisfaction(%)				180.115	*p* < 0.01
Yes	1,852(85.6)	1,605(90.3)	247(63.8)		
No	312(14.4)	172(9.7)	140(36.2)		
Residence(%)				0.287	0.633
Urban	758(35.0)	627(35.3)	131(33.9)		
Rural	1,406(65.0)	1,150(64.7)	256(66.1)		
Number of CMDs				10.365	0.001
2 ~ 3	1,919(88.7)	1,594(89.7)	325(84.0)		
4 ~ 5	245(11.3)	183(10.3)	62(16.0)		
Age				−6.582	*p* < 0.01
60 ~ 69	1,330(61.5)	1,144(64.4)	186(48.1)		
70 ~ 79	670(31.0)	525(29.5)	145(37.5)		
≥80	164(7.6)	108(6.1)	56(14.5)		
Child satisfaction(%)				10.979	0.04
Yes	1,834(84.8)	1,526(85.9)	308(79.6)		
No	248(11.5)	192(10.8)	56(14.5)		
No child	82(3.8)	59(3.3)	23(5.9)		
Distant object vision(%)				−3.684	*p* < 0.01
Good	463(21.4)	396(22.3)	67(17.3)		
Common	1,018(47.0)	851(47.9)	167(43.2)		
Bad	683(31.6)	530(29.8)	153(39.5)		
Near object vision(%)				−0.548	0.583
Good	516(23.8)	436(24.5)	80(20.7)		
Common	1,088(50.3)	876(49.3)	212(54.8)		
Bad	560(25.9)	465(26.2)	95(24.50)		
Hearing(%)				−1.375	0.169
Good	561(25.9)	461(25.9)	100(25.8)		
Common	1,150(53.1)	961(54.1)	189(48.8)		
Bad	453(20.9)	355(20.0)	98(25.3)		
Memory(%)				−1.9	0.057
Good	255(11.8)	207(11.6)	48(12.4)		
Common	1,024(47.3)	865(48.7)	159(41.1)		
Bad	885(40.9)	705(39.7)	180(46.5)		
Education(%)				−0.609	0.542
Below junior high school	1,857(85.8)	1,529(86.0)	328(84.8)		
High school or vocational school	244(11.3)	195(11.0)	49(12.7)		
College or above	63(2.9)	53(3.0)	10(2.6)		
Financial resources after retirement(%)				1.232	0.540
Children	1,154(53.3)	957(53.9)	197(50.9)		
Savings or pension	913(42.2)	740(41.6)	173(44.7)		
Else	97(4.5)	80(4.5)	17(4.4)		
Marriage(%)				4.787	0.091
Married	1,686(77.9)	1,400(78.8)	286(73.9)		
Divorced or widowed	459(21.2)	361(20.3)	98(25.3)		
Unmarried	19(0.9)	16(0.9)	3(0.8)		
Night sleep duration	6(5,8)	6(5,8)	6(5,8)	−0.774	0.439
ADL score	6(6,6)	6(6,6)	5(3,6)	−13.498	*p* < 0.01
Income	5,000(60,30,801)	5,000(80,30,020)	5,765(−2,431,35,000)	−0.596	0.551
Cognitive function	10(6,13)	10.5(7,13.5)	7(3,11.5)	−9.116	*p* < 0.01
BMI	25(22,28)	25(22,27)	24(18,29)	−2.712	*p* < 0.01

### 3.2 Screening predictor variables using LASSO and binary logistic regression

**Figure 2:**
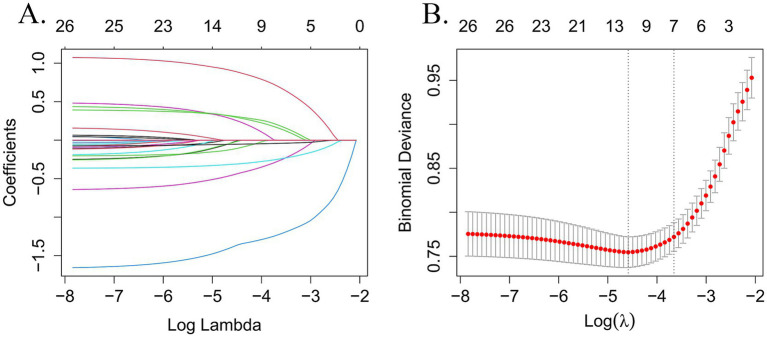
Perform LASSO regression on 26 independent variables. **(A)** Coefficient path diagram for 26-variable LASSO regression. **(B)** The cross-validation curve for LASSO regression uses the optimal penalty coefficient (*λ*) as the final selection criterion.

In this study, LASSO regression was conducted on the training set using the “glmnet” package in R software. The analysis yielded an optimal lambda value of 0.0102 and identified 11 significant predictors of frailty (see Figures 2A,B). These predictors were subsequently incorporated into a binary logistic regression model using SPSS for further analysis. The final results indicated that factors such as depressive symptoms, drinking habits, history of falls, life satisfaction, ADL scores, cognitive function, and age were significantly linked to the risk of frailty in older adult CMM patients (see Table 3).

**Table 3:** Binary logistic regression analysis.

Variable	*β*	SE	Wald χ^2^	OR	*p* value	95%CI
Depression						
No	Reference					
Yes	0.377	0.173	4.759	1.483	0.029	1.039 ~ 2.046
Social activity						
No	Reference					
Yes	−0.631	0.163	15.086	0.532	<0.001	0.387 ~ 0.731
Fall down						
No	Reference					
Yes	1.066	0.160	44.492	2.905	<0.001	2.124 ~ 3.974
Life satisfaction						
No	Reference					
Yes	−1.704	0.203	70.158	0.182	<0.001	0.122 ~ 0.271
Number of CMD						
2 ~ 3	Reference					
4 ~ 5	0.479	0.229	4.376	1.614	0.036	1.031 ~ 2.529
Age						
60 ~ 69	Reference				<0.001	
70 ~ 79	0.392	0.170	5.298	1.480	0.021	1.060 ~ 2.066
≥80	0.956	0.261	13.450	2.602	<0.001	1.561 ~ 4.337
ADL score	−0.364	0.047	60.603	0.695	<0.001	0.634 ~ 0.761
Cognitive function	−0.072	0.019	14.170	0.931	<0.001	0.896 ~ 0.966

### 3.3 Development of predictive models

**Figure 3:**
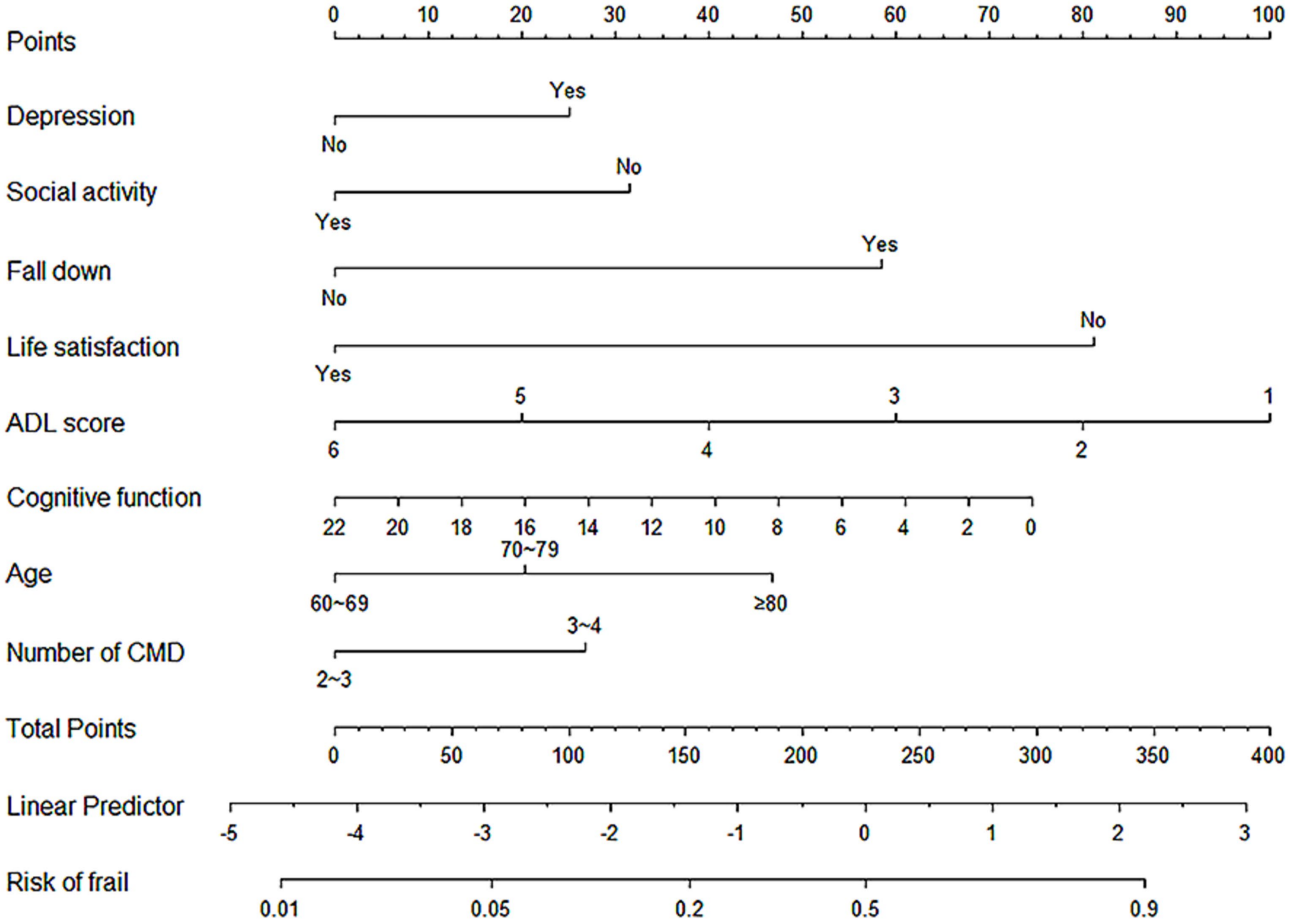
Nomogram for Predicting Frailty in Older Adult CMM Patients.

After identifying the risk factors for frailty in older adult Chinese CMM patients, this study constructed a binary regression model using the “rms” package and the lrm function in RStudio. The final model included depression, social activity, history of falls, life satisfaction, ADL scores, cognitive function, age, and the number of CMDs as predictors (see Figure 3). A nomogram was constructed to provide a quantitative prediction of frailty risk in older adult CMM patients. The scores for each predictor were derived from the upper portion of the model, and the likelihood of frailty was assessed by plotting a vertical line between the total score axis and the associated risk axis.

### 3.4 Validation of the prediction model

#### 3.4.1 Differentiation

**Figure 4:**
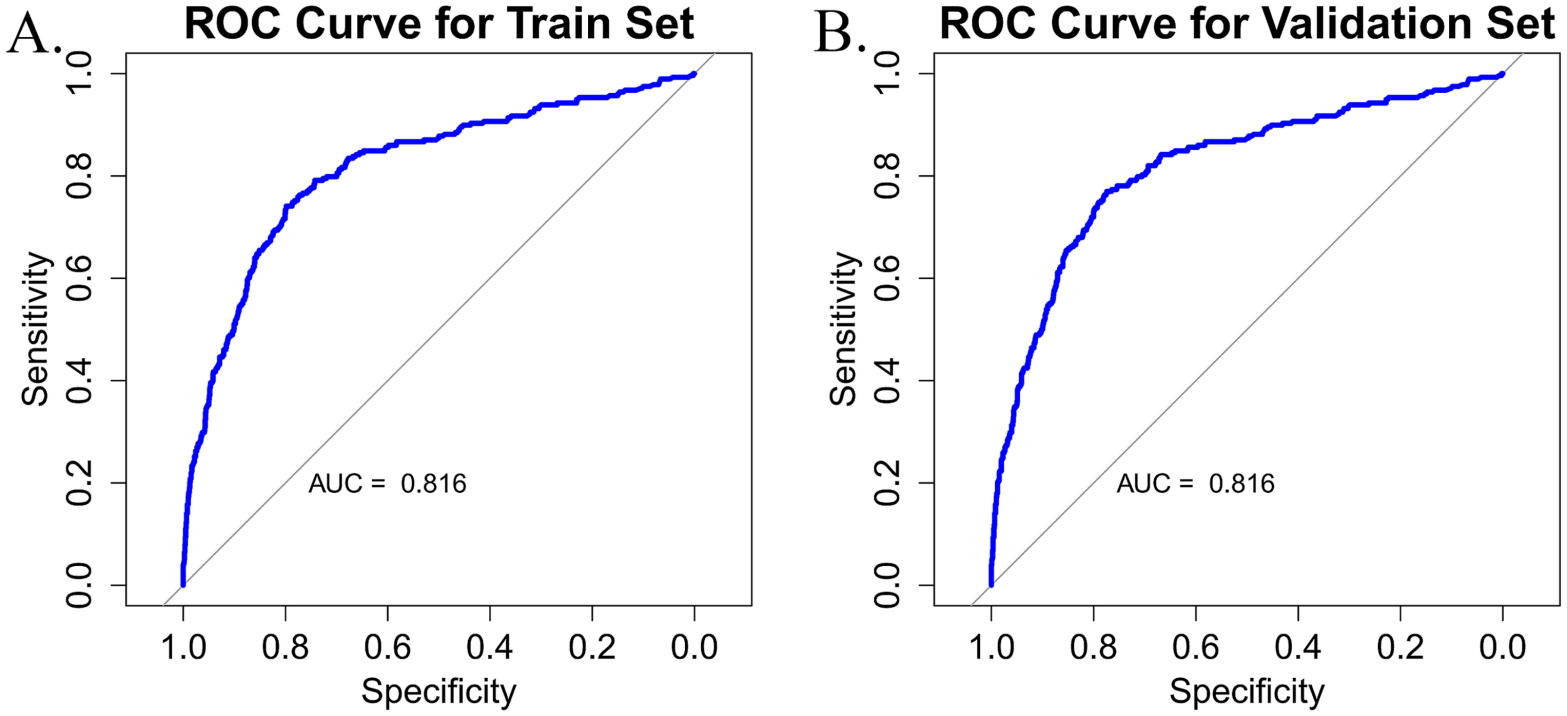
**(A)** Nomogram ROC curve generated from the training dataset. **(B)** Nomogram ROC curve generated from the validation dataset.

The discriminatory performance of the predictive model was evaluated by calculating the AUC values for both the training and validation datasets. As shown in Figures 4A,B, the model exhibited a good performance in both sets. In the training set, the AUC was 0.816 (95% CI: 0.787–0.848), while in the validation set, the AUC was 0.816 (95% CI: 0.786–0.846). Both the lower and upper limits of the AUC were greater than 0.5, with all values exceeding 0.7, indicating that the model has strong discriminatory power and can effectively distinguish between frail and non-frail individuals among older adult CMM patients. The model has significant clinical value for early screening and intervention, offering essential support for frailty management and the promotion of health among older adults.

#### 3.4.2 Calibration

**Figure 5:**
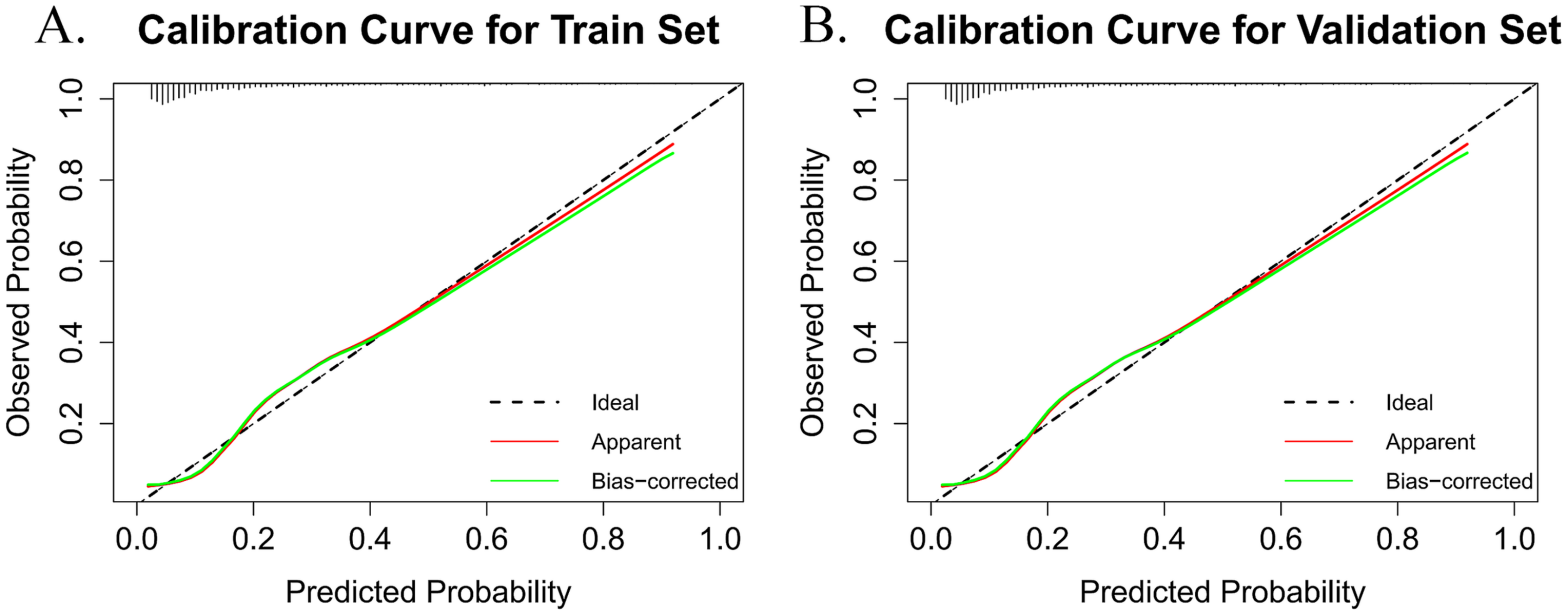
The black dashed line (ideal) represents the theoretical reference line when predictions are completely accurate. The red solid line (apparent) is the calculated calibration curve. The green solid line (bias-corrected) is the calibration curve after bias correction. **(A)** Calibration plot for the train dataset. **(B)** Calibration plot for the validation dataset.

The accuracy of the nomogram was evaluated using the Hosmer-Lemeshow goodness-of-fit test and calibration plots, where a *p*-value > 0.05 indicated a well-calibrated model. The findings demonstrated that the model performed admirably for both the training set (χ^2^ = 8.812, df = 3, *p* = 0.073) and the validation set (χ^2^ = 10.288, df = 8, *p* = 0.245). The calibration plots for the training set (Figure 5A) and the validation set (Figure 5B) closely approximated the ideal curve, highlighting an excellent agreement between the predicted probabilities and the actual likelihood of frailty. Therefore, this model can be applied not only to individual patient health management but also as a valuable predictive tool for public health agencies. By forecasting frailty, public health institutions can implement targeted intervention strategies to reduce frailty prevalence among older adults and enhance their overall quality of life.

#### 3.4.3 Clinical validity

**Figure 6:**
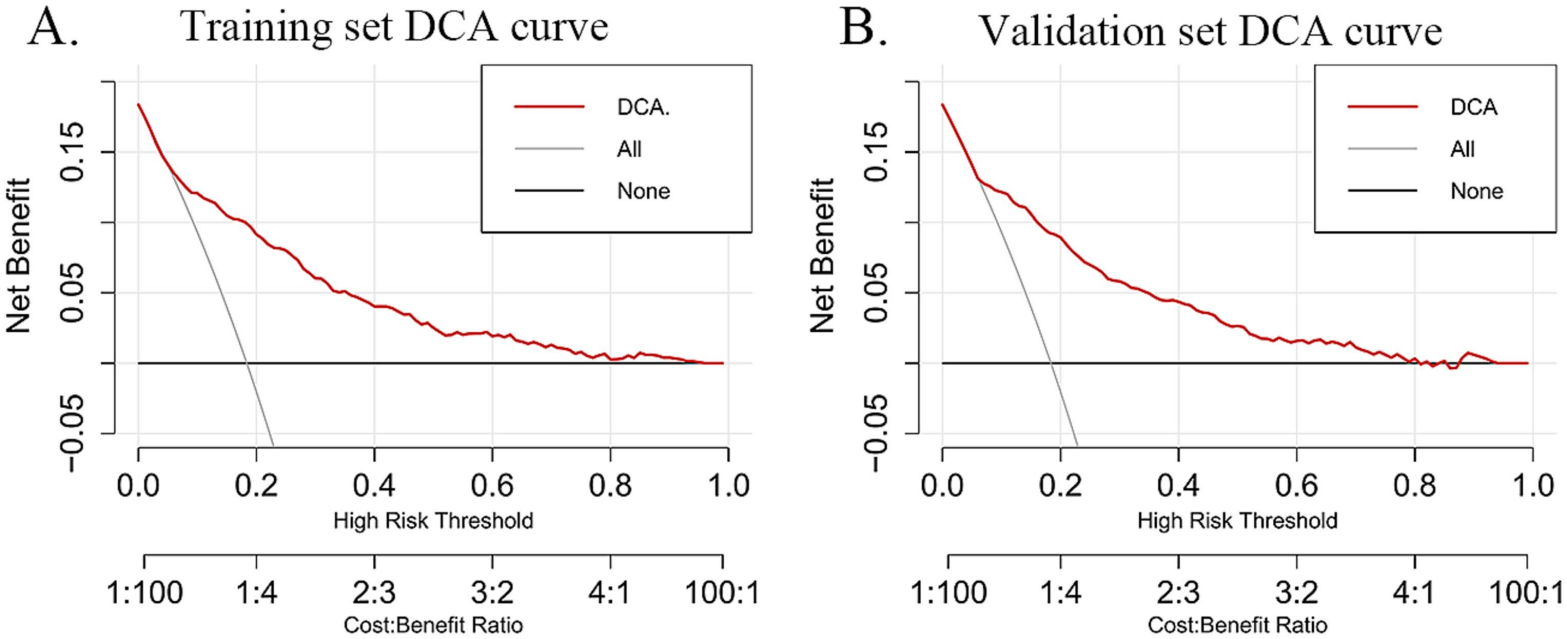
The red curve (DCA) represents the decision curve based on the model, illustrating the net benefit of the model under different cost-benefit ratios as the high-risk threshold varies. The slash gray curve (All) represents the “treat all” strategy, showing the net benefit of treating all individuals at different high-risk thresholds. The black line (none) represents the “treat no one” decision strategy and depicts the net benefit of this approach across different high-risk thresholds. **(A)** DCA curves for the training dataset. **(B)** DCA curves for the validation dataset.

The DCA was utilized to evaluate the practical value of the nomogram in clinical decision-making. The nomogram model demonstrated a superior net benefit in both the training set (Figure 6A) and the validation set (Figure 6B) compared to the “treat-all” and “treat-none” strategies. This finding suggests that the model serves as a valuable decision-making tool for predicting frailty risk in older adults with CMM, offering strong clinical applicability and practical utility. Furthermore, it exhibits robust predictive accuracy and clinical effectiveness in assessing frailty risk within this population.

#### 3.4.4 Subgroup analysis

This study conducted a subgroup analysis on categorical variables, including age, depression, social activity, fall history, life satisfaction, and the number of CMDs. The detailed results are presented in Table 4. All AUC values were greater than 0.7, indicating that the model demonstrated consistency and excellent predictive performance across different settings. Notably, the model exhibited better predictive performance in individuals aged 70–79 years, those with depression, and those with a higher CMD count. Therefore, the model exhibits good applicability across different populations.

**Table 4:** Subgroup analysis.

Variable	AUC	95%CI	Sensitivity	Specificity
Age				
60 ~ 69	0.775	0.725 ~ 0.825	0.672	0.856
70 ~ 79	0.855	0.817 ~ 0.893	0.889	0.699
≥80	0.730	0.634 ~ 0.827	0.600	0.816
Depression				
Yes	0.808	0.771 ~ 0.844	0.851	0.647
No	0.764	0.706 ~ 0.823	0.712	0.780
Social activity				
Yes	0.788	0.730 ~ 0.846	0.742	0.781
No	0.799	0.761 ~ 0.838	0.729	0.750
Fall down				
Yes	0.799	0.756 ~ 0.842	0.792	0.666
No	0.777	0.728 ~ 0.825	0.701	0.790
Life satisfaction				
Yes	0.772	0.730 ~ 0.814	0.671	0.803
No	0.729	0.662 ~ 0.796	0.610	0.822
Number of CMDs				
2 ~ 3	0.807	0.774 ~ 0.841	0.767	0.753
4 ~ 5	0.851	0.783 ~ 0.919	0.913	0.678

#### 3.4.5 Confusion matrix analysis

**Figure 7:**
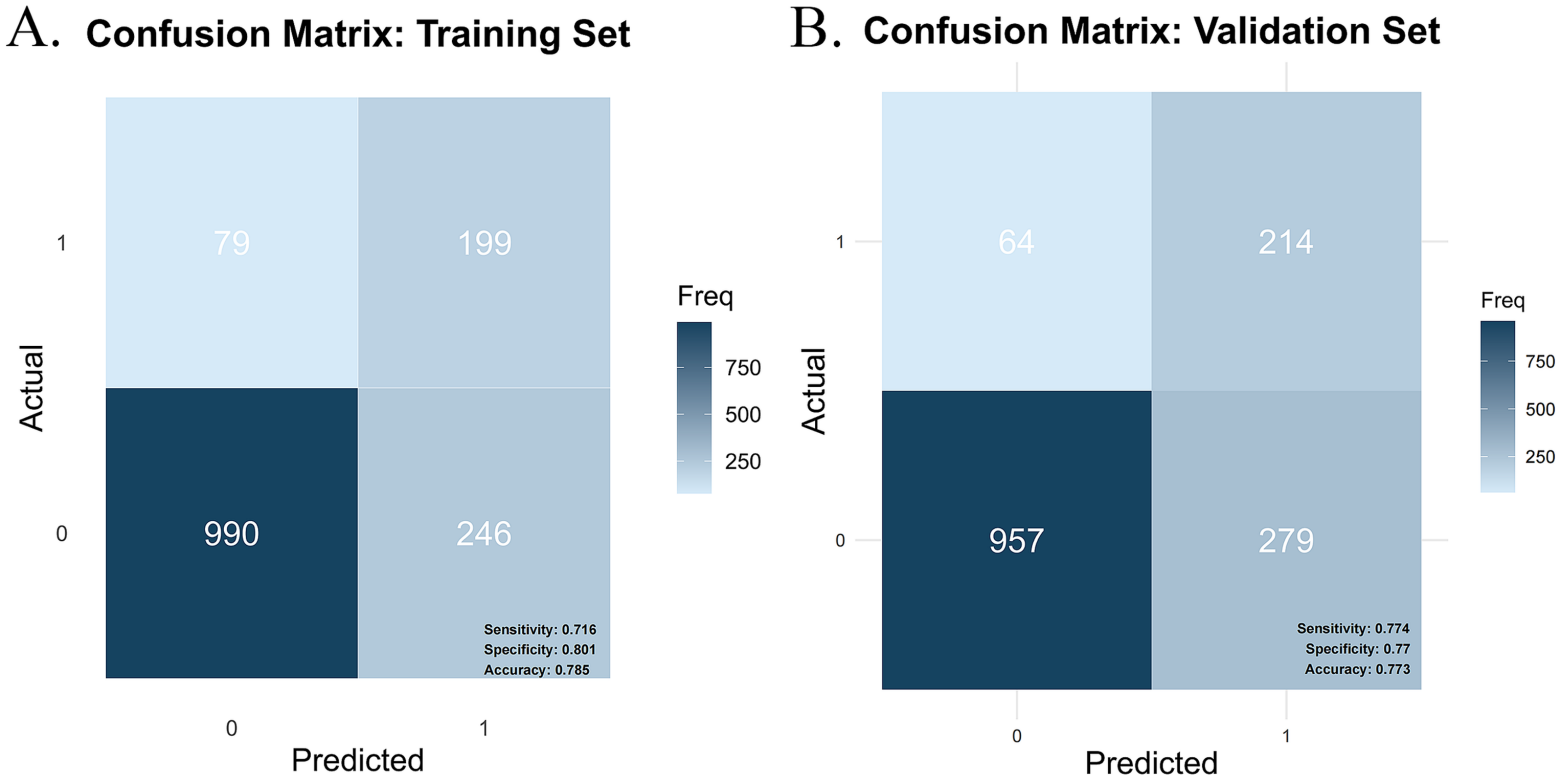
Confusion matrix plot. **(A)** Training set confusion matrix. **(B)** Validation set confusion matrix.

In this study, the confusion matrices for both the training and validation sets (Figures 7A,B) illustrate the predictive performance of the model in frailty identification. In the training set, the model achieved a sensitivity of 0.716, a specificity of 0.801, and an overall accuracy of 0.785. While in the validation set, the model demonstrated a sensitivity of 0.774, a specificity of 0.770, and an overall accuracy of 0.773. These results indicate that the model exhibits strong predictive capability in both the training and validation datasets, effectively distinguishing between frail and non-frail individuals within the older CMM population. Moreover, the balanced sensitivity and specificity across both datasets further reinforce the model’s reliability and stability, supporting its suitability for real-world clinical applications.

## 4 Discussion

This study adopted a cross-sectional design and analyzed large-sample data from 2015 on older CMM patients in China to examine the prevalence and influencing factors of frailty in this population. Additionally, a nomogram was utilized to predict frailty risk. This study not only provides a new perspective for understanding the frailty risk among older CMM patients but also lays a solid theoretical foundation for developing targeted prevention and intervention strategies. Furthermore, it holds significant clinical application value in frailty prediction. The observed frailty prevalence of 17.88% aligns closely with the 16% prevalence reported in a meta-analysis by Vertrano et al. (33) for older adult individuals with multimorbidity. Moreover, this prevalence exceeds the frailty rate observed in patients with a single CMD (10.1%) (26) within the same database using the same frailty measurement tool, as well as the overall frailty prevalence among older adults (8.87%). These results indicate that the incidence of frailty is higher among individuals with CMM than among those with a single CMD and the general older adult population.

In comparison to previous studies, the present research found that factors such as sex, educational level, marital status, residence, and sleep duration had relatively minor effects on frailty in older adult patients with CMM. This result differs slightly from studies that focus on individuals with a single CMD and the general older adult population. The differences between this study and previous research may stem from variations in the frailty assessment tools employed, as well as the higher proportion of rural residents (65%) in this cohort. Additionally, the sample size and the population’s heterogeneity may partially explain these differences. Unlike studies that broadly examine the total number of chronic diseases, this study specifically focuses on individuals with a high prevalence of CMM, providing a more targeted approach by accounting for the number of CMDs conditions they have. This study, which encompasses a nationwide sample, is larger in scale and more representative. The nomogram developed in this study identifies key factors associated with frailty in older adult CMM patients, including depression, social activity, falls, life satisfaction, ADL scores, cognitive function, and age.

Existing research suggests that depression and frailty share similar pathological mechanisms (34). In this study, depression and social inactivity were identified as significant risk factors for frailty in older adult CMM patients, aligning with findings from Deng et al. (35) and Hanlon et al. (36). Depressive symptoms have been shown to correlate with elevated concentrations of inflammatory markers including C-reactive protein (CRP), interleukin-6 (IL-6), and tumor necrosis factor-alpha (TNF-*α*), which can impair muscle strength and function, manifesting as diminished grip strength and slowed walking speed (37, 38). Depression may also disrupt the neuroendocrine system, leading to dysfunction of the hypothalamic–pituitary–adrenal axis (39). As a negative mental health condition, depression can reduce interest in eating and social activities, increasing the risk of malnutrition and physical inactivity, both of which contribute to frailty (40). Social activity, which involves meaningful interactions and engagement, plays a crucial role in maintaining both physical and mental health. Given the growing prevalence of frailty, it is essential for clinicians to assess the level of social activity in older adult patients and implement tailored interventions to address this aspect of their care (41).

ADL is an essential measure for evaluating a person’s capacity to carry out daily living activities independently. The predictive model in this study shows a significant link between low ADL scores and frailty among older adult CMM patients. Previous research has established a robust correlation between frailty and physical functional decline, further validating ADL as a key predictor of frailty (42). Low ADL scores not only reflect a decline in self-care ability but may also adversely affect dietary habits, increasing the risk of malnutrition. Furthermore, physical functional decline is frequently coincides with lower activity levels, leading to diminished muscle strength and reduced bone density, thereby increasing the risk of sarcopenia and osteoporosis-two conditions that significantly contribute to frailty (43). Clinicians should systematically assess patients’ ADL scores to identify frailty risk early on. Implementing integrated interventions can help older adult patients maintain or improve their self-care capabilities, preventing further deterioration of frailty, and enhancing their overall well-being.

The predictive model in this study reveals that older adult patients with CMM and lower cognitive function scores exhibit significantly higher levels of frailty compared to those with higher scores, indicating that cognitive impairment is a critical risk factor for frailty. This finding is consistent with the results reported by Ma et al. (44). Cognitive impairment can lead to brain gray matter atrophy and neurodegenerative changes, which cause autonomic nervous system dysfunction and accelerate frailty progression (45, 46). These neurophysiological changes hinder patients’ ability to communicate and access social resources, impairing their capacity to obtain and process information. Consequently, this negatively affects social interactions and reduces the likelihood of seeking social support (47). Additionally, cognitive impairment often coexists with physical functional decline and limitations in daily activities, resulting in significantly reduced social engagement. This gradual social disconnection can lead to feelings of loneliness and helplessness, exacerbating psychological and emotional distress, creating a vicious cycle, and severely affecting patients’ quality of life and overall health (48). Moreover, studies have shown that an increased number of chronic multimorbidities correlates with poorer cognitive performance (49). The combined effect of multiple conditions may accelerate cognitive decline through synergistic interactions. Incorporating cognitive function assessments into routine frailty evaluations for CMM patients allows healthcare providers to achieve more accurate risk stratification and ensures that patients receive timely, targeted interventions to address both cognitive and physical health challenges.

Age is a crucial factor influencing frailty in older adult CMM patients, with frailty prevalence increasing markedly with advancing age (28). Aging is linked to heightened concentrations of inflammatory markers and a gradual dysregulation of immune mechanisms. Research indicates that immune dysfunction and chronic inflammation are critical pathogenic factors in the development of frailty (50). Chronic inflammation adversely affects skeletal muscle mass and strength in older adults by inhibiting protein synthesis and increasing protein degradation. Inflammatory biomarkers have been closely linked to frailty and mortality risk in older adults (41). Additionally, older adults with CMM often experience a gradual reduction in daily activity participation due to declining physical capacity and the loss of labor roles. After retirement, their social interactions may become limited, further weakening their social function and roles (51). Therefore, for older populations, particularly those at higher risk, early intervention and targeted care are essential. Such measures can significantly improve their quality of life, delay the progression of frailty, and reduce the risk of complications.

Life satisfaction, a critical aspect of subjective well-being, represents an individual’s assessment of their quality of life according to personally defined standards. As a cognitive assessment of life, it is often associated with emotional experiences (52, 53). This study finds that life satisfaction as a protective factor against frailty in older adults, supporting the findings reported by Wilhelmson et al. (54). However, this finding contrasts with studies on individuals with a single CMD and those with disabilities, in which life satisfaction did not reach statistical significance as a predictor of frailty (26, 27). This discrepancy may be attributed to the complexity of the CMM population, which is characterized by multimorbidity and interrelated physiological and psychological responses. In contrast, life satisfaction among individuals with a single CMD or disabilities may be more strongly influenced by a single disease or non-health-related factors, potentially reducing its predictive significance in frailty models. These differences underscore the importance of considering individual differences and population-specific characteristics in frailty prediction. As an important indicator of mental health and social well-being, life satisfaction plays a critical role in preventing and mitigating frailty progression in older adults. Higher life satisfaction is typically associated with positive emotional states, strong social support networks, and greater psychological resilience. These factors work together to support physical function and overall health in older adults, emphasizing the significance of enhancing life satisfaction in this group.

The study also found that older adult patients with CMM who had a history of falls were 2.458 times more susceptible to frailty than those with no such history. Evidence suggests that higher frailty indices are associated with an increased likelihood of falls, with a bidirectional relationship existing between the two (33, 55). However, this predictive factor differs from findings in studies on frailty among individuals with a single CMD and the general older adult population. To our knowledge, previous frailty studies based on the CHARLS database have not included fall history as a predictive factor. In contrast, our study incorporated fall history based on expert consultation and identified it as a significant predictor of frailty in older adults with CMM. This finding may be attributed to the fact that falls can result in physical injuries, such as fractures, which significantly limit mobility and increase the duration of bedrest, thereby accelerating musculoskeletal decline-a primary pathological mechanism underlying frailty (56). Additionally, approximately 20%–40% of individuals who experience falls develop a fear of falling (39). This psychological barrier often leads to excessive restriction of physical activity, resulting in social isolation, reduced psychological resilience, and a cascade of adverse outcomes that further exacerbate the onset and progression of frailty. These findings underscore the importance of comprehensive health assessments for older adult individuals, particularly those with a history of falls. Healthcare providers should evaluate not only frailty status but also fall risks. Particularly for high-risk older CMM patients with a history of falls, timely and targeted fall rehabilitation training should be implemented to reduce the risk of falls and prevent the vicious cycle of frailty caused by falls.

According to the binary regression results, the probability of frailty in CMM patients with 4–5 CMDs is 1.614 times higher than in those with 2–3 CMDs. An increasing number of CMDs is associated with a higher likelihood of frailty, a finding that aligns with previous research (21). Vinjerui et al. (57) conducted a study on older adults in Belgium, examining the relationship between multimorbidity, functional dependence, and frailty. The study found that frailty mediates the association between the number of chronic diseases and functional dependence, with all three factors being interrelated, mutually influential, and overlapping to some extent, while also serving as predictors of one another. The comorbid state of older adults with CMM heightens their vulnerability, and when chronic conditions are not effectively managed over time, the likelihood of frailty development increases. This underscores the need for targeted vigilance in individuals with multiple CMDs and emphasizes the importance of enhancing screening efforts for CMM patients to identify those at risk of progressing to frailty, thereby enabling timely interventions.

Frailty is a multidimensional process influenced by complex factors, making it challenging to achieve optimal outcomes through a single intervention. Therefore, public health policies should first prioritize early screening for older adults with CMM, particularly among high-risk frailty populations, by conducting comprehensive assessments of factors such as depression, social participation, falls, and cognitive function to establish a foundation for timely intervention. Second, personalized health management plans should be implemented based on specific frailty risk factors. For example, individuals with low life satisfaction and pronounced depressive symptoms should be prioritized for psychological interventions, while those with low ADL scores may benefit from targeted functional rehabilitation training. Finally, public health strategies should holistically consider the eight factors identified in the nomogram and adopt a multidimensional, interdisciplinary intervention approach. For instance, among older adults with CMM, integrating psychological health support, social activity promotion, fall prevention, and cognitive function training can effectively mitigate the onset and progression of frailty.

This research has several limitations. First, the data were derived from a Chinese sample, which means that the model is primarily applicable to Chinese populations. Its external validity requires further evaluation using data from other countries to assess its applicability and generalizability across diverse cultural and socioeconomic contexts. Second, the CMD disease information in the database is based on self-reports rather than biochemical measurements, which may introduce recall bias. Finally, frailty in older adult patients with CMM is a complex and multidimensional process influenced by various factors, including dietary habits, medication use, and other relevant factors. However, due to limitations in the CHARLS database, this study was unable to comprehensively capture all potential contributing factors. Therefore, future research should be explored in greater depth from the following three perspectives. First, additional variables, such as medication use and dietary habits, should be further investigated and incorporated to gain a more comprehensive understanding of the underlying causes of frailty. Expanding the range of predictive variables could enhance the accuracy of the predictive model and improve its clinical applicability. Second, this study is based on a Chinese database and employs a cross-sectional research design. Future studies should incorporate data from different countries and adopt a longitudinal study design to analyze the dynamic progression of frailty and examine how various factors influence its development over time. Such an approach would allow for a more comprehensive evaluation of the relationship between CMM and frailty while also facilitating the exploration of commonalities and differences across diverse healthcare systems and national contexts. Finally, although this study has developed a comprehensive predictive model, clinicians often require more practical and accessible tools in real-world applications. Future research should focus on developing simplified and user-friendly frailty prediction tools based on artificial intelligence techniques such as machine learning. Additionally, comparative analyses of multiple predictive models should be conducted to identify the optimal model, thereby improving predictive performance and enhancing its practicality and efficiency in clinical and public health decision-making.

## 5 Conclusion

This study incorporated depression, social activity, fall history, life satisfaction, ADL scores, cognitive function, age, and the number of CMDs into the nomogram model to predict frailty risk in older adult CMM patients. The evaluation of the model’s discrimination, calibration, and clinical utility demonstrated strong performance in terms of discriminatory power, calibration accuracy, and clinical applicability. Consequently, the nomogram model developed in this study serves as a theoretical foundation for improving health management in this population and offers scientific support for the development of effective prevention and intervention strategies.

## Data Availability

Publicly available datasets were analyzed in this study. This data can be found at: http://CHARLS.pku.edu.cn.
