# Supplementary material for: Development and validation of a predictive model for frailty risk in older adults with cardiovascular-metabolic comorbidities
Source: Front Public Health. 2025 Apr 22;13:1561845. doi: 10.3389/fpubh.2025.1561845 (PMC12052544; doi:10.3389/fpubh.2025.1561845)
Supplement: Supplementary file 1 [file Supplementary_file_1.docx]

Supplementary Material

# Supplementary Figures and Tables

## Supplementary Figures**
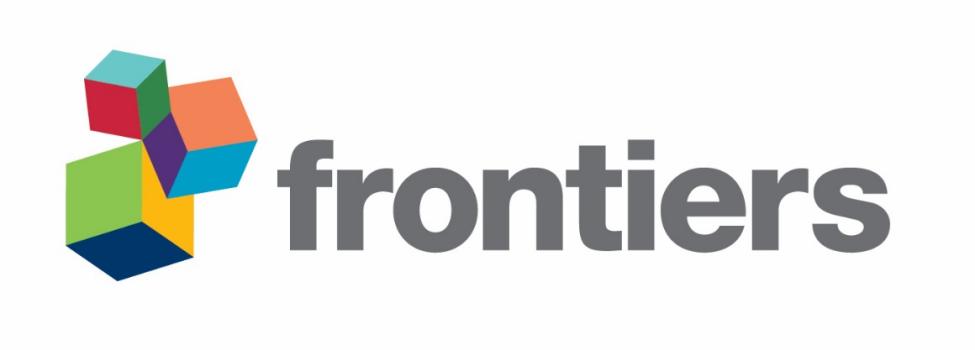
**

**
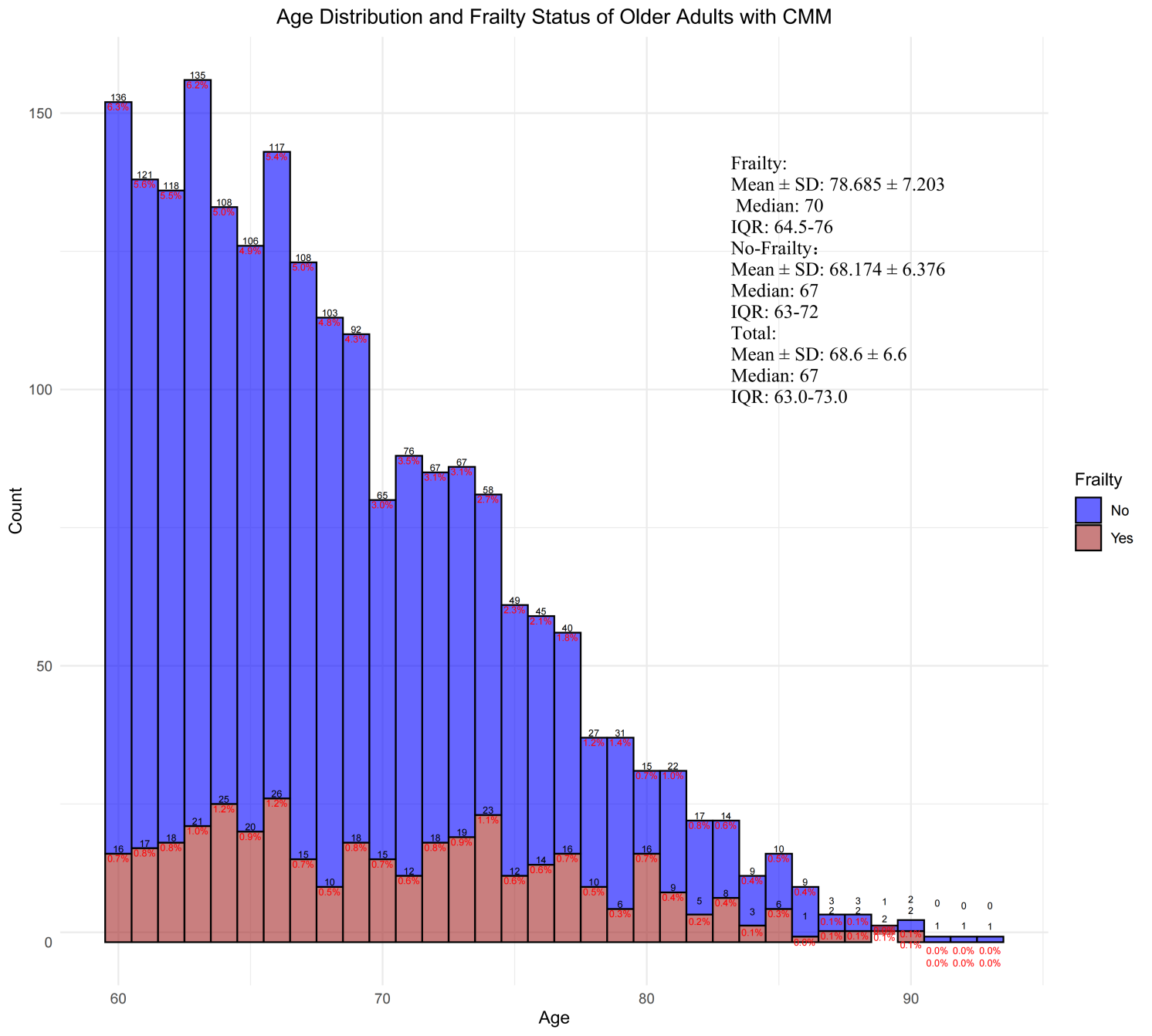
**

**Supplementary Figure 1.** The age distribution chart of older CMM patients and the frailty status chart at each stage, where each histogram represents one year. Mean ± SD represents the mean plus or minus the standard deviation, while IQR represents the interquartile range.


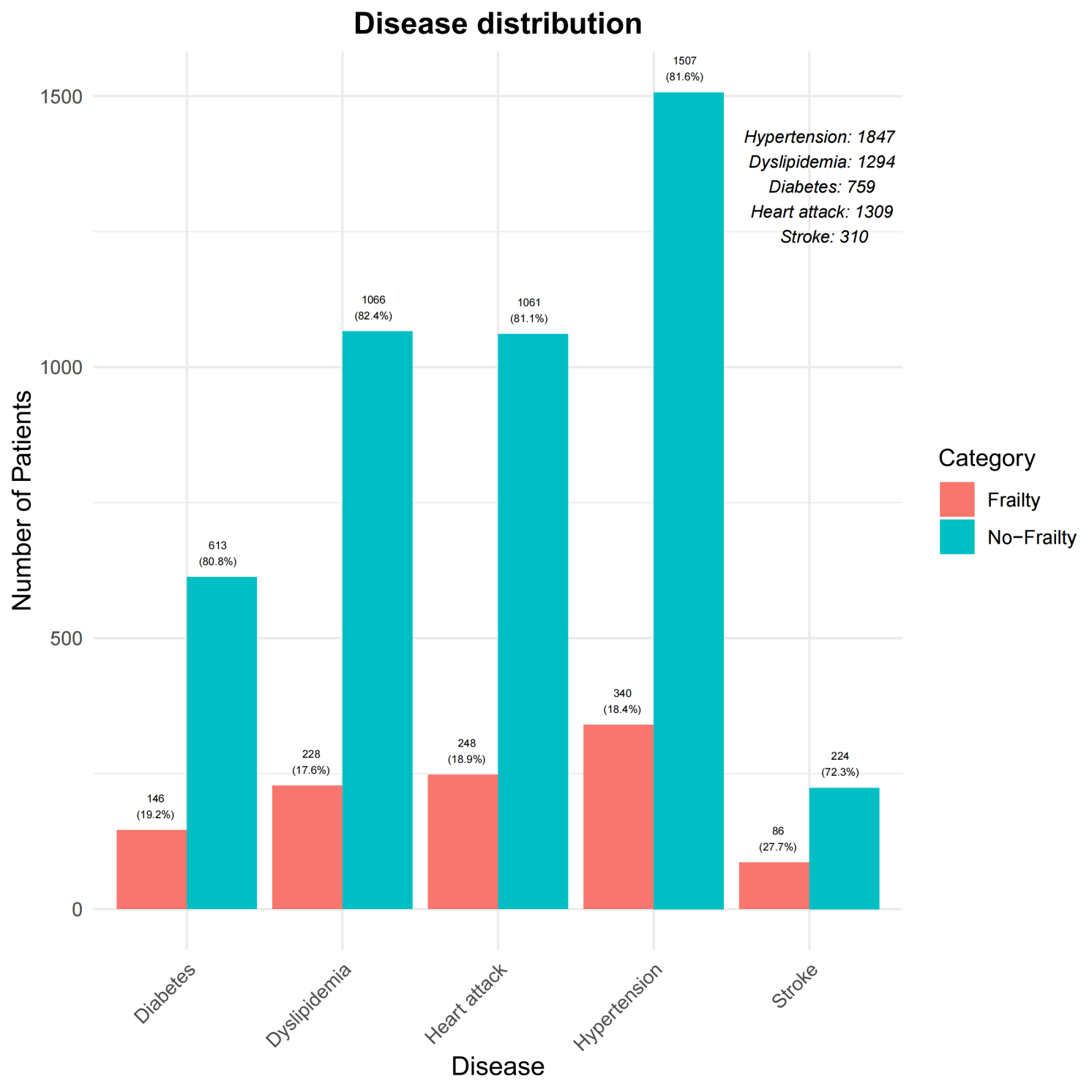


**Supplementary Figure 2.** The disease distribution chart illustrates the frailty distribution within each disease and the number of patients for each disease.

**
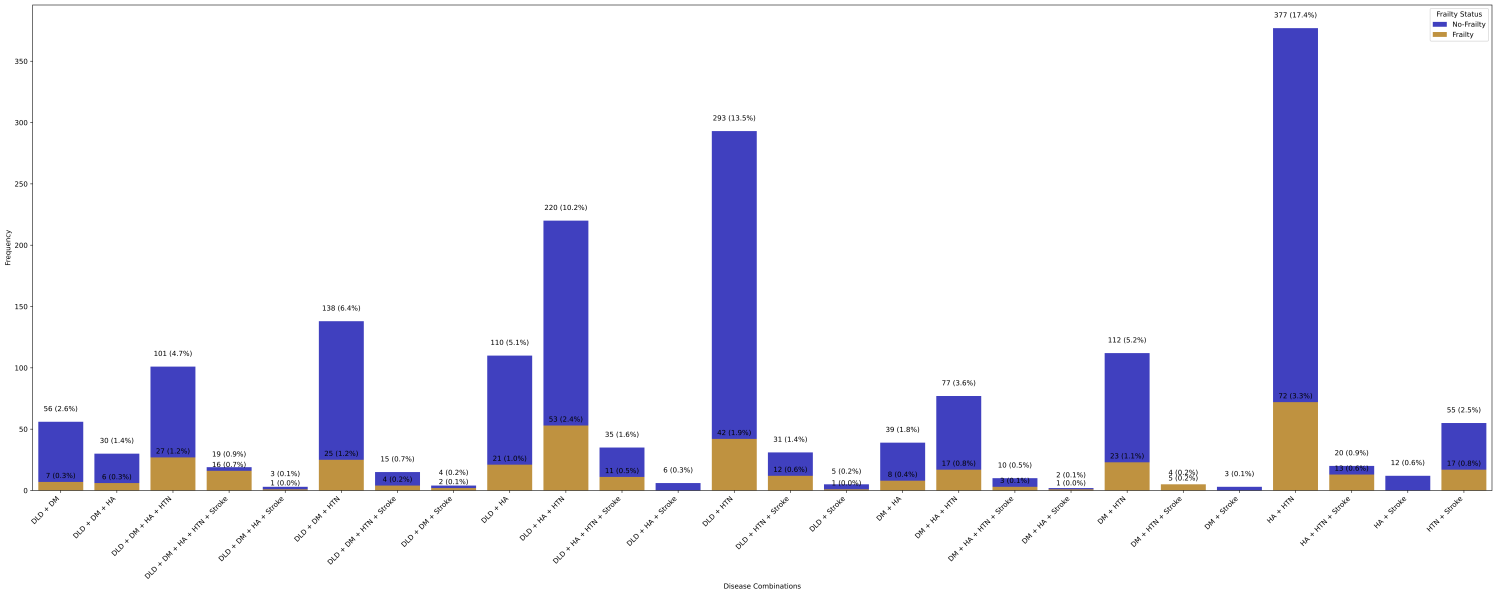
**

**Supplementary Figure 3.** The different CMD comorbidity patterns in CMM patients, where DLD represents Dyslipidemia, DM represents Diabetes, HA represents Heart Attack, and HTN represents Hypertension.


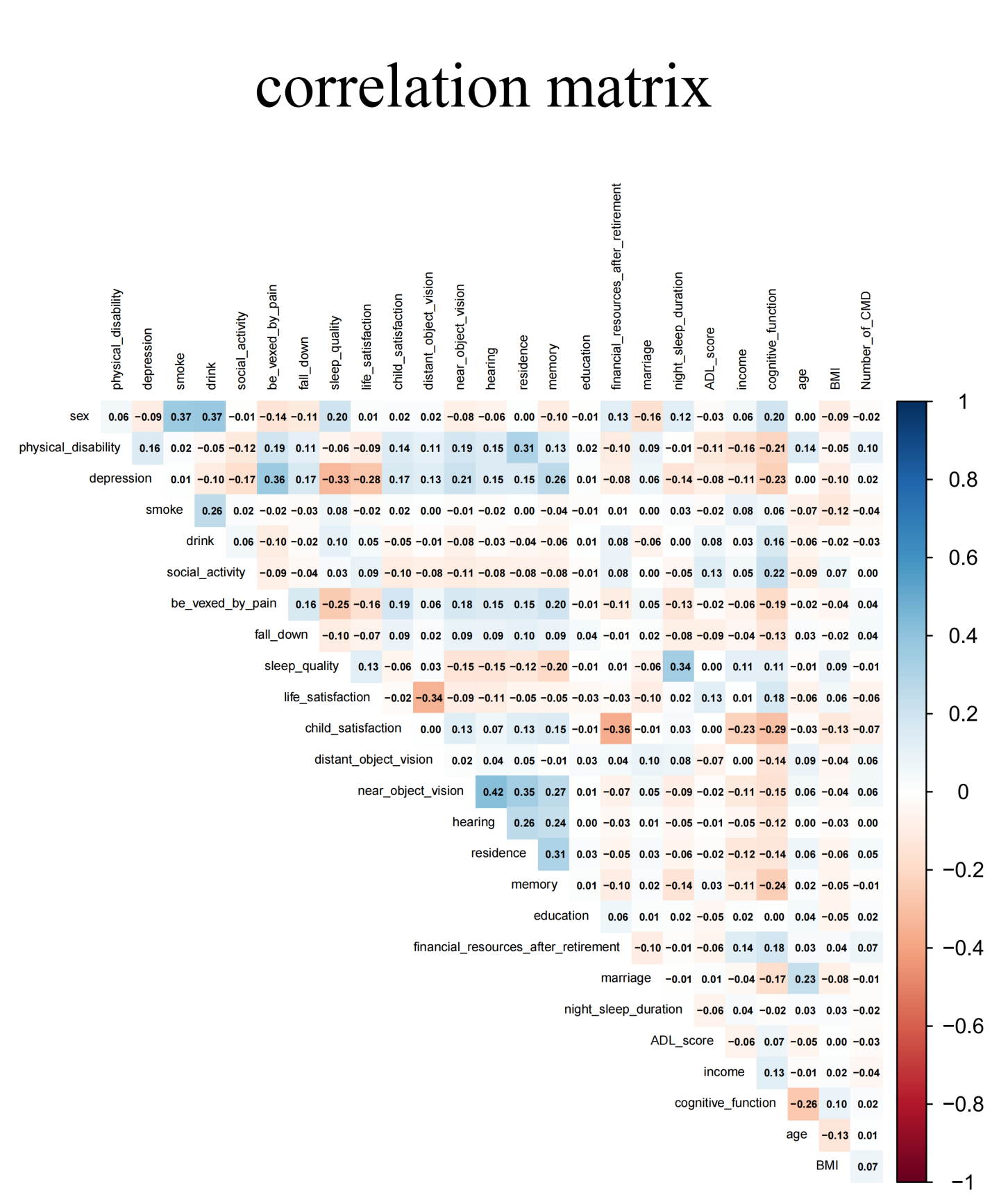


**Supplementary Figure 4.** Correlation Matrix Distribution Plot of 26 Predictive Variables.
